# A survey of barriers and facilitators to primary care staff conducting research

**DOI:** 10.1371/journal.pone.0334892

**Published:** 2025-11-25

**Authors:** Zoe Edwards, Michael Tatterton

**Affiliations:** 1 School of Pharmacy & Medical Sciences, University of Bradford, Bradford, West Yorkshire, United Kingdom; 2 Affinity Care Primary Care Network, Bradford, West Yorkshire, United Kingdom; 3 School of Nursing & Healthcare Leadership, University of Bradford, Bradford, West Yorkshire, United Kingdom; Lamar University, UNITED STATES OF AMERICA

## Abstract

**Background:**

Research is essential to find ways of improving healthcare although is not established in primary care. This study aims to investigate the barriers and facilitators to primary care staff conducting research in one geographical area of the UK.

**Method:**

A survey was developed for primary care staff using validated questions where possible. It was distributed using Online Surveys^TM^ by email, social media and personal contact.

**Results:**

One hundred and ten participants were recruited from 29 GP practices in one geographical area, 74 (67.2%) were female and 35 (31.8%) were male. Nearly three quarters (73.6%) were clinical with the remainder non-clinical staff. Sixty percent of participants had been asked to conduct research and just over 40% had never conducted research. Twenty-three participants (20.9%) had conducted research six or more times. The largest facilitator for research was the knowledge that it would make a difference to patients. Allocated time, clear communication about what was involved and making a difference for colleagues were also ranked highly. The largest barrier for research was the inability to fit anything extra in and concerns over how much work would be involved. Thirty-nine percent of clinicians knew how to gain knowledge from evidence and apply it to practice. Those who were enrolled on an educational course were more likely to be research active.

**Conclusion:**

Primary care staff are more likely to take part in research if it has clear patient benefit. Communication about time commitments, research outcomes and data security are important when deciding to become involved. Staff may worry that they do not have the right skills to take part. Recommendations include co-design of studies, improved communication and administrative support. The recording of research activity and literacy at clinician annual review would allow these parameters to be measured.

## Introduction

The pressures on the National Health Service (NHS) are greater than they have ever been. In a post COVID-19 world there are backlogs in healthcare and resources are increasingly stretched to cope with demand [1]. The reasons for this are complex and plentiful. COVID-19 led to delays in diagnosis and treatment for cancer and long-term conditions leading to a population which now has a greater need for care [1]. COVID-19 itself led to its own set of long-term conditions which are wide-ranging and require new resource which was not previously required [1]. It also led to a decrease in the population’s mental health and a deterioration in other conditions related to weight and alcohol consumption influenced by repeated lockdowns [2]. Even before the pandemic, the NHS was under increasing pressure for staff and resources [3]. In the absence of greater funding, finding better ways of working, more effective medicines and treatment pathways to lessen the strain on the NHS - by research - is the only way of relieving this pressure.

Since 2019, GP practices were encouraged to work together in Primary Care Networks (PCNs) to pool resources and specialisms to increase access to high quality healthcare [4,5]. Approximately 99% of GP practices are now in PCNs and these often serve between 30,000 and 50,000 patients, sometimes more [6].

Practices or PCNs are sent details of studies recruiting in the area, but it is up to them whether they conduct the research. Invitations usually come via email and when these emails are received, they are often passed on to other staff with specific skills or roles who are asked to conduct the necessary activities. The research requested of primary care comes in many forms. They may act as a Participant Identification Centres (PIC) and will be asked to do searches to find patients with certain conditions and then invite those who are eligible. The research may be testing an intervention and more complex in-depth involvement is required perhaps requiring additional training. Specific research studies may sometimes ask staff for interviews about specific issues regarding patients with certain disease groups (such as dementia) and how their activity is adapted to help those patients. Increasingly, primary care is being asked to conduct more complex research, sometimes in the form of late stage drug trials and there are national funding streams to encourage participation [7]. For these trials clinicians are involved in testing new medicines or technologies within a primary care setting by either referring identified patients to research teams or indeed, testing the new medicines and technologies with patients themselves. Historically, it was only GPs who were asked to take part in primary care research but now all staff can be asked, not just GPs. It is increasingly common for other clinicians, administrative staff and managers to be asked to take part.

The traditional model of primary care being delivered by doctors and nurses has changed. As there are an ever-increasing number of patients living with an increased number of complex conditions, the health service needs to expand to include more healthcare professionals from different backgrounds. The Additional Roles Reimbursement Scheme (ARRS) was brought in to support this and can include roles such as social prescribers and care coordinators therefore increasing the diversity of roles in primary care [8]. Many new roles are from Allied Healthcare Professions (AHPs) such as paramedics, podiatrists and physiotherapists as well as from other professionals such as nurses and pharmacists. These professions were not traditionally as involved in research although the NIHR has identified that they have a role to play with increasing funding opportunities for non-medics [6]. Research has been found to be difficult to incorporate into medical, non-medical and emerging roles even when it is a requirement [9]. This is perhaps a reflection of the stress in the system in an overburdened NHS.

In preparation for this work, a systematic review was carried out looking at the work that had been done into the barriers and facilitators to staff taking part in primary care research [10]. Each study showed barriers and/or facilitators to conducting research in a particular study. Results showed lack of time, skills, knowledge and funding as well as more surprising findings of concerns around ethics, lack of support and poor communication.

### Research question

What are the barriers and facilitators to primary care staff taking part in research in one geographical area in the north of England.

## Method

A survey was chosen to find the barriers and facilitators of primary care staff taking part in research as it was timely, easy to distribute and has the ability to collect data from a large volume of participants [11]. The associated systematic review found that time was the most commonly cited barrier to research found therefore using a quick and easy to complete collection tool would help to ensure as many views as possible are obtained [10]. A survey can also explore whether the barriers and facilitators found in the literature are applicable in the population of interest in the post-COVID-19 timeframe.

### Ethics

Ethics permission was granted by the Chair of the Humanities, Social and Health Sciences Research Ethics Panel at the University of Bradford on 8^th^ August 2023.

### Survey design

The survey was developed from other similar surveys conducted with healthcare professionals around the world and based on the findings from the associated systematic review [10] (S1 Appendix). Validated questions were used where available and appropriate, and information was taken from other research findings and adapted to suit the aims of this study [12,13]. Input was requested at several stages from the local ICB, the Clinical Research Network (CRN), clinicians and administrative staff to ensure findings were as useful as possible and had the potential to improve participation. It was piloted with a group of clinical and non-clinical staff and changes were made according to feedback. Changes included the inclusion of extra job roles, changes in formatting and improvements in the clarity of the questionnaire. The piloting process showed that the average time for completion was five minutes.

The survey began by asking the first part of the postcode of the practice in which the participant works. The purpose of this was to link the answers to the research activity in that area although all answers were confidential.

The main part of the questionnaire was for all staff. This collected demographic data, research activity and barriers and facilitators to research. Clinicians were asked to complete a small extra section of questions and were directed via the survey mechanism to do this depending on the answers they gave about job role.

### Survey distribution

The Online Surveys^TM^ platform was used to enable links to be sent to participants. This platform was free to access and distribute and allowed multiple question types and qualitative extensions to answers where applicable. The survey was open from 11^th^ August 2023–22^nd^ September 2023 inclusive. Participation was voluntary and consent was inferred by online written survey completion which was authorised by the ethics committee. Weighted practice recruitment tables were provided by the Clinical Research Network (CRN) showing recruitment based on the complexity of each study (ranging between 0–1720 patients recruited and weighted recruitment values of 0–2233). These were divided arbitrarily into high research activity (200–3000 weighted recruitment per year), medium research activity (20–199 per year) and low (0–19 per year). The two practices from the top, two middle and two bottom practices in terms of research activity were identified and targeted in person. Personal visits to the practices were requested and offers to present at training meetings were made. This method was adopted so practices with high, medium and low research activity were included. All other practices in the geographical area were invited to take part in the study via ICB distribution by email, social media and word of mouth.

### Setting

The setting being explored was that of staff working in the primary care setting in one geographical area of the UK. This area is a diverse area including inner city and rural populations in the north of England. The local ICB was involved in identification of practices and distribution to known practice contacts.

Participants were identified from the practices as outlined above providing they adhered to the inclusion and exclusion criteria below.

#### Inclusion criteria.

Any administrative, managerial, or clinical staff employed in primary care within one ICB area aged over 16 years old.

#### Exclusion criteria.

Anyone working outside the ICB area

Anyone under 16 years old

Any cleaning or maintenance staff who would be unlikely to be asked to take part in research

### Sample size

The ICB area includes 648000 people and 67 practices [14]. Estimates of typical staffing levels in primary care show that this population level would typically include 410 GPs, 162 Nurses and 702 administration staff [15]. This makes the potential population and sample size 1274. Unfortunately, there are no more up to date sources including newer roles such as AHPs and Practice Pharmacists. In a similar study targeting 46000 primary care professionals, with some targeted recruitment, 414 questionnaires were returned giving a response rate of 0.9% [16]. This recruitment rate would give us an expected sample size of 35. A mailed survey in Germany had a recruitment rate of 37% however it involved a substantial payment for participation and this study does not [12].

### Recruitment

Practices were emailed by the ICB and the researcher and the survey distributed by online link. A Quick Response (QR) code was available for use when presenting to groups of staff for immediate access. The survey was also distributed via social media networks of the researcher, publicly available emails and sharing was encouraged. Participation was voluntary. The survey was available for completion for six weeks due to the time restrictions on the study and although no email reminders were sent, the survey was advertised on social media networks multiple times. Participants did not have to answer every question in the survey, and they did not have to complete the ranking questionnaires in full with only four responses needed to carry on to the next question (this was the level set by the online tool). The contact details of the researcher and their academic supervisor were available to participants for them to ask any questions they had.

### Data analysis

Data were extracted into Excel spreadsheets and analysed by frequency. Free text responses were grouped or added to any multiple-choice answers accordingly. Data are summarised descriptively. Ranking questions were divided into what participants had ranked as the five most important barriers or facilitators and the five least important barriers and facilitators and put into tables (see S1 Appendix).

## Results

Staff were recruited from 29 different GP practices throughout the geographical area being studied. This represents 43% of the total number of practices. None of the practices contacted chose to take the researcher up on their offer of a short, personalised presentation or a one-to-one conversation about the research other than the PCN where the researcher was employed (from which there were 4 practices and 65 responses). This practice was in the top level of research activity as per the CRN data discussed earlier.

One hundred and ten people provided responses to the survey giving an estimated response rate of 8.6%. Thirty (27.0%) participants were non-clinical staff and the remaining 81 (73.6%) were clinical staff. See Table 1 for the breakdown of the results.

**Table 1:** Results of the primary care survey.

	Total
Total participants	110
Female	74 (67.2%)
Male	35 (31.8%)
Role – non-clinical	
Administrative (including care navigation)	18 (16.3%)
Manager	12 (10.9%)
Role – clinical	
Advanced Clinical Practitioner	11 (10.0%)
Healthcare assistant	6 (5.4%)
GP locum	0
GP partner	21 (19.0%)
GP salaried	19 (17.2%)
GP trainee	1 (0.9%)
Nurse	9 (8.1%)
Pharmacist	8 (7.2%)
Physician Associate	3 (2.7%)
Other	3 (2.7%)
Asked about conducting research	
Asked in an administrative role	18 (16.3%)
Asked to be interviewed	34 (30.9%)
Asked to be involved	37 (33.6%)
Asked to complete a survey	46 (41.8%)
Not asked	44 (40.0%)
Unsure	1 (0.9%)
If asked, how many times	
1-2 times	14 (12.7%)
3-5 times	25 (22.7%)
6 or more times	27 (24.5%)
How many times have you taken conducted research	
1-2 times	22 (20.0%)
3-5 times	20 (18.2%)
6 or more times	23 (20.9%)
Not taken part	44 (40.3%)
Research as part of appraisal (clinician only)	
Yes, routinely	6 (7.4%)
Sometimes	19 (23.5%)
Only if I bring it up	21 (25.9%)
No	27 (30.0%)
I haven’t had an annual review	8 (9.9%)

Of the 76 clinicians who completed the survey, 29 (38%) had been qualified between 11–20 years and 31 (40.8%) had been qualified over 21 years. Nine (11.8%) had been qualified up to 5 years and 11 (14.5%) between 6–11 years. Of all participants, 40 (36%) had been in their current role more than 10 years, 13 (11.7%) 5–10 years, 24 (21.6%) 3–5 years, 21 (18.9%) 1–2 years and 13 (11.7%) under 12 months. Sixty-six (59.5%) of participants had a postgraduate degree and 11 (9.9%) were enrolled in further or higher education courses at the time of the survey including PhD and independent prescribing courses. This information was collated from the free-text responses.

Sixty-six (60%) participants had been asked to conduct research in some form and the remaining 44 participants (40%) had never been asked to. Sixty-five of the 109 people who answered the question said they had conducted research one or more times.

**Fig 1:**
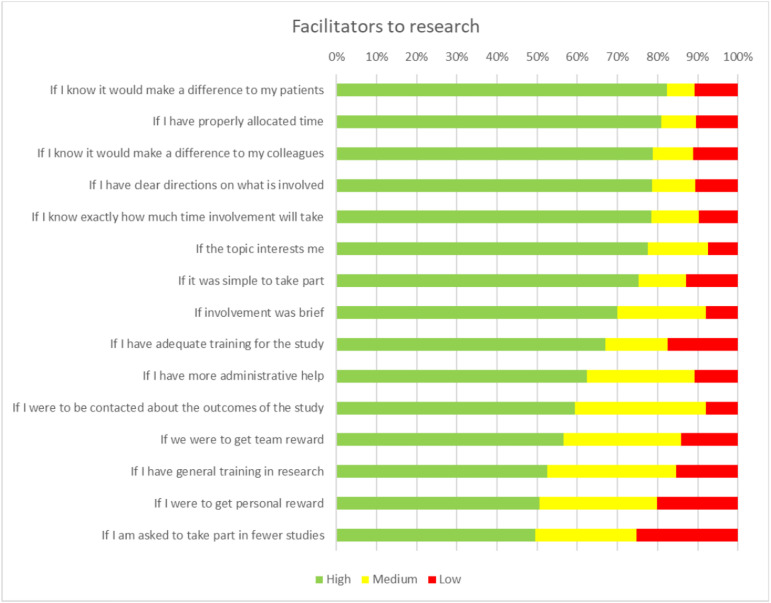
A chart showing the facilitators to research ranked as high, medium and low importance by general practice staff.

Fig 1 shows the facilitators to research ranked by the primary care staff. Differing numbers of participants answered each question (range 97–107) so numbers are expressed as a percentage of those who answered. There were 15 facilitators in total which were ranked as 1 being the most important and 15 the least important. The largest facilitator (where 82.4% of participants ranked it between 1 and 5) was the knowledge that research would make a difference to their patients. This was closely followed by allocated time (81%), knowledge that it would make a difference to colleagues (78.8%) and clear directions on what was involved (78.6%) and certainty as to how much time it would take (78.4%).

**Fig 2:**
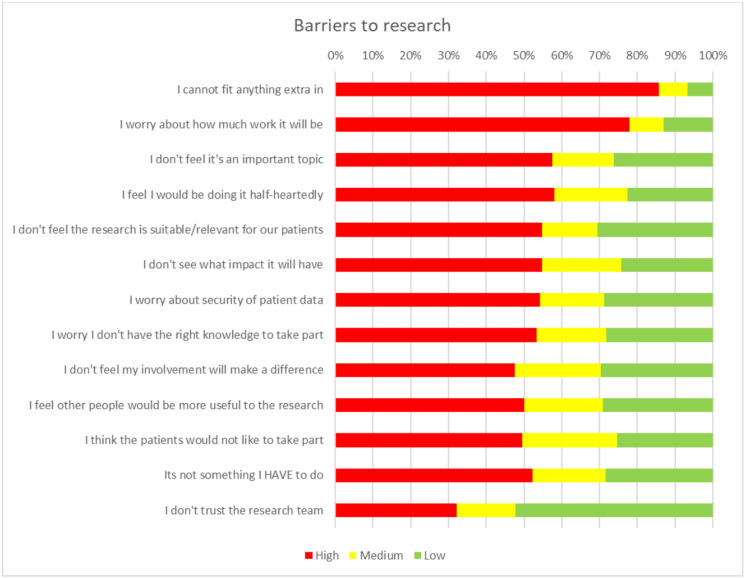
A chart showing the barriers to research ranked as high, medium and low importance by general practice staff.

Fig 2 shows the 14 barriers to research as ranked by the primary care staff where the biggest barrier was ranked highest. The largest barrier was that staff could not fit anything extra in (85.7%), followed by concerns about quantity of work (78.0%). Although not seen as one of the biggest barriers, lack of trust in the research team was ranked between 1 and 5 by 32.2% of participants.

**Fig 3:**
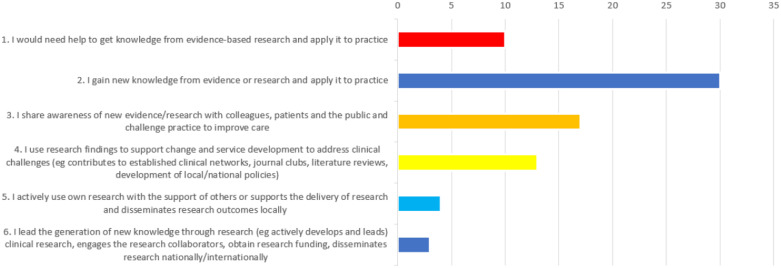
Achart showing clinician’s self-declared research involvement where 1 is the least and 6 is the greatest.

Fig 3 shows the self-declared research involvement of clinicians in the study. Thirty participants (39%) felt they gained new knowledge from evidence or research and applied it to practice although 10 (12.9%) would need help to do this. Four (5.2%) participants actively used their own research and 3 (3.9%) felt they lead new generation of knowledge through research.

Of the 11 participants who responded that they were enrolled in an educational course, 10 completed details. Table 2 shows the research activity of those participants and self-declared research involvement of clinical participants. The self-declared research involvement is shown as numbers one to six (as in Fig 3) for ease.

**Table 2:** A table showing the clinical participants who were enrolled in a further or higher education course and their research activity and self-declared involvement.

Current role	Been asked to take part in research	Number of times taken part in research	Self-declared research involvement
Manager	Yes	6+	4
ACP	Yes	3-5	3
Pharmacist	Yes	6+	3
Manager	Yes	1-2	n/a
Pharmacist	Yes	6+	6
Other	No	0	2
ACP	No	0	3
ACP	No	0	2
PA	Yes	3-5	4
ACP	Yes	6+	4

## Discussion

This survey showed that staff are more likely to take part in research when they feel it is relevant to both patients and colleagues. Communication, which was simple, clear and succinct, was a facilitator to staff becoming involved in research. Time restraints were the largest barrier with other issues relating to time and effort being evident. Most clinicians surveyed knew how to access evidenced-based research and apply it to their practice, but few were involved in the generation of new knowledge.

The findings of this study show the importance of simple, efficient and relevant research being carried out in primary care by staff who have adequate time and support to carry it out. The response rate of the survey was better than other similar studies which did not provide financial incentives to participate [16].The survey results indicate that everyone who has been asked to take part in research has, at some point, taken part as the number who had never been asked and had never taken part were the same.

The largest facilitator found was the knowledge that research would make a positive difference to patients which was not evident in the themes of our systematic review [10]. Two American studies found that improved patient outcomes and relevance to patients were motivators to take part, but these were not explored further possibly due to studies looking at staff involvement rather than that of patients [17,18]. Previous surveys and interviews demonstrated a lack of patient-centredness about thoughts surrounding research with relevance to the interests of practices seen as the important factor rather than how the research may be able to help their patients [19–23]. This may be due to the increase in focus on person-centred care in the UK over the last few years and much of the literature being relatively old [24]. This study shows that staff are more likely to take part if there is an obvious route to patient benefit. A simple way to address our findings would be clear communication of patient benefit when talking to general practice staff about research studies. This would have to be balanced against providing too much information to staff to maintain their need for succinct communication.

The largest barrier to research was time, with staff saying they cannot fit anything extra into their working weeks. Properly allocated time for research was an important facilitator for participants as found in previous studies where staff may not start exploring research without funded time [17,18,20,22,23,25–36]. The literature found that even when clinicians wanted to be part of research, it sometimes felt like a choice between clinical care and research with the latter often being carried out in staff’s own time [20,27]. Conversely, our survey showed that properly allocated time for conducting research would be a facilitator. Currently, as seen in the literature, research is not part of the primary care contract and is rarely something which is expected of practices. Therefore, it is understandable that there is no allocated time for this activity with so many other competing priorities such as patient care, Quality Outcomes Framework (QOF) activities and administration.

Workload concerns were also found to mirror findings from previous studies despite the majority of previous studies being carried out in times where the health service was under less pressure than it is at present

[37]. Over three-quarters of survey participants placed high importance on the accuracy of the suggested time it would take to complete the required task for the research and a similar number placed high importance on the clarity of what would be involved when making their decision. This finding echoes the importance of timely and accurate communication and realistic expectations being needed from the researchers for staff to be able to carry out research [21,26,34]. Often estimates of time requirements for research are wide ranging and inaccurate with the individual members of staff feeling the burden of assessing how much time may be needed [21,26]. This can lead to clinicians being put off further research involvement after a bad experience and the loss of potential interest and expertise [33]. This could be addressed by University or Industry researchers piloting the amount of time for involvement in the study and giving clear and accurate details of what is involved when research is communicated.

Concerns about training and knowledge were important to participants with just over half of participants placing high importance on the statement that they feel they do not have the right knowledge to take part. Previous studies have shown staff with more experience and training in research were more likely to be more positive about future involvement [25]. This is difficult to address as any additional training would require a greater time commitment from a population who already feel they do not have adequate time. Language used in research may be off-putting to staff and make them feel like they may not know enough to take part [17]. Clear communication of requirements for research or subject knowledge when communicating would ensure staff had more confidence that they were the intended audience.

Although not the largest barrier, a significant proportion of participants worried about security of patient data with over half placing this as high importance. Importance of trust issues with the researchers was ranked highly by nearly a third of participants. This echoes findings in other studies. In the UK, research has to go through a strict ethical process before approval which is rigorous, lengthy and detailed [38]. Patient information is either anonymised or kept securely and deleted after a set period of time [38]. Staff in primary care may not be aware of this process so training or communication about this may go some way to increasing understanding and confidence in the process so they are more trusting of researchers [17,19].

Communication about research outcomes was also a facilitator for research with the majority of participants ranking this highly. This finding has been seen previously and it had the potential to incentivise future research participation as participants feel their involvement was worthwhile [17,26]. This is simple to address through communication to participants who wish to be informed of study results after completion.

Participants thought the availability of administrative help was important when choosing whether to take part in research with nearly two-thirds ranking this as of high importance. Research in primary care often involves non-clinical duties and seemingly unnecessary administration which clinicians may have to do, taking precious time away from their core roles and adding to work pressures [22,31,36,39]. Investment in non-clinical research administration roles would free up clinical time to take part in complex studies [20]. Furthermore, co-design of studies at an early stage between academia, industry and primary care may allow optimisation of research processes to reduce unnecessary steps making it easier to take part [26,39].

As research is an optional activity in primary care it was perhaps not surprising that team or personal reward were ranked highly by approximately half of the participants. Levels of recompense are not likely to be known by many staff although they will likely be aware that it may not be enough to cover their time. Although team incentives (such as providing lunch) may be a short-term way to improve engagement, it may not work in embedding research practice in primary care culture. Funding and recognition was a theme in the systematic review although not in the same context [10]. Poor funding was a barrier but recognition in the form of recognised professional development or further job opportunities may go some way to facilitating research [17,18,20,29,33].

Research findings pertaining to the clinicians self-declared research involvement aimed to find out how research was used in the individuals practice and was based on a tool developed in a previous study used recently to measure views of Allied Healthcare Professionals (AHPs) in the UK [13,40]. Results of this study show the population surveyed to be at a lower research literacy level compared with the AHPs in the 2022 study. Comer [13] showed that over half of participants were in the top three research levels (four to six) compared with only a quarter in this study and the AHP study had around a fifth in the top two levels (five and six) compared with less than a tenth in this study. This difference may be due to the distribution of the Comer [13] survey being mostly through research channels and perhaps attracting those who were more engaged with research already. Alternatively, those surveyed in the Comer [13] study were all AHPs working in any part of the NHS whereas this section of this study surveyed all healthcare professionals working in general practice. In this study, those who were enrolled in educational courses had a higher self-declared research involvement than those who were not as over a third declared they were in the highest three research levels. Of those who were doing educational courses, several were Advanced Clinical Practitioners for which there is a mandatory research element, so it was therefore surprising that only half of them had been asked or taken part in research activity. Postgraduate qualifications often include research training and the literature also found this to be a facilitator as staff have a greater understanding of research and have an easy source of information for any problems they may encounter [25,32].

Although not the highest ranked question; more than half of participants placed high importance on the fact that research was not something they had to do as part of their role. Only a very small proportion of participants routinely discussed research as part of their appraisal. To the author’s knowledge this question has not previously been asked in research studies however this is in line with previous findings where primary care research was not seen as something which would improve career progression so therefore may not be core to the role [23,31,32]. Several previous studies recommended that management take research more seriously and it should be supported from the top down in organisations [28,32]. Another study not included in the associated literature review (as it did not meet the eligibility criteria) recommended that research visibility be improved at national, organisational, team and individual levels with suggestions including discussions at appraisal and specific clinical academic roles [13]. Comer [13] used the self-declared research interest scale with AHPs however this could be something that could be used more widely among all staff within primary care to gauge and perhaps even encourage their research involvement. The current promotion of clinical academic roles by the NIHR goes some way to addressing this for those who wish to go down the route where some of their time can be put aside for research and paid accordingly [41].

## Recommendations

Many of the barriers reported by participants cannot easily be addressed. The time and workload pressures of the NHS in present times are not things that are likely to subside quickly. Fair recompense for time taken in primary care to complete research activity is also a bigger problem which may need further insight and input from research support networks. There are, however, positive recommendations which can result from this survey which will help address the barriers and facilitators identified. Also, a measure of research activity by staff would be useful to identify trends in research as well as literacy of evidence-based medicine. Table 3 shows recommendations as a result of this study.

**Table 3:** Recommendations for future research for primary care.

Recommendations
1. Pilot involvement in research and provide timings, duties and required knowledge with suggested staffing in communication.
2. All studies involving primary care should be developed alongside partners working in the field to ensure research is appropriate, efficient and well-designed.
3. Communication with primary care should succinctly state how patients will benefit from the study.
4. All communication with staff inviting them to take part should clearly state that the research has been through a rigorous ethics process and any data will be kept secure.
5. Research findings should routinely be fed back to participating sites
6. Research literacy and involvement information should be routinely measured for all clinicians as part of the annual appraisal process.
7. Funded research administration roles to be considered.
8. Communication about studies with primary care should be clear, easy to read and efficient.

## Strengths and limitations

The strength of this research was that a systematic literature review was done before the questions were developed and questions were based on findings from this previous literature [10]. Where possible, validated questions were used. The ICB and the CRN were invited to contribute to and comment on the concept and the draft survey to ensure as wide a range of viewpoints were included as possible to strengthen the study and make the results as useful as possible. The response rate was higher than expected and included many practices throughout the area although the majority of participants were doctors, so a completely balanced viewpoint was not gained. Although participants were from 21 different practices, 65 of those were from one PCN with already high research activity where the researcher worked (as this was the only one allowing a personal visit) so was known to participants and this may have led to bias in the results. The survey software used allowed participants to answer questions they had been asked not to (due to job role) therefore future surveys may require upgrades to ensure participants only answer the questions appropriate for them. The aim of this study would have been more comprehensively answered through qualitative interviews, but this method was not possible within the timescales of this work.

Although survey questions were derived from data found in the qualitative systematic review, this also can be a limitation. Ranking questions may have omitted barriers and facilitators as they were not included or reported on. The majority of papers in the systematic review were qualitative interviews or focus groups so this should minimise the chances of unknown barriers or facilitators being reported on.

A limitation of the research was that following the piloting of the survey, changes were made to the layout of the multiple answer questions. Although these were re-piloted, this was accessed via personal computers with large screens. In practice, the adapted multiple answer questions were difficult to read on a smartphone screen where many participants accessed the survey. This may have led to fewer participants completing the survey. The research tool used was an online survey which required a level of computer literacy to use. This may have prevented views being captured from less computer literate staff although a certain level of information technology knowledge is assumed for employees in general practices.

The reliability of the survey may be affected by non-response bias as we do not have information about staff who did not respond although this is a common feature of surveys [11]. It is possible that non-responders would have different barriers and facilitators to responders but these are unclear from these results and could be addressed through future qualitative interviews with staff.

Only one geographical area was surveyed meaning that responses from other areas were not captured and may differ to those found in this study. Results may differ in areas with different research activity or with more or less support from their CRN. There was an innate bias in this survey as those who had strong feelings or those who were more used to taking part in research were more likely to take part.

The author has a background in academic health research and currently works in primary care as an ACP, Research Lead and Regional Pharmacy Lead for NIHR. This background and role allows insight into research from both sides which, although not unique, is very unusual. The intention was that this insight would strengthen this research but it may also lead to the authors biases and personal opinions influencing both the literature review and the interpretation of the survey.

## Further research

Future research in this area as part of a team approach would strengthen its findings and allow exploration and dialogue of findings amongst the research team rather than having only one viewpoint. Further research could include qualitative interviews with staff to further explore barriers and facilitators to research. Additional statistical analyses could be carried out to assess statistical significance of findings. Our methods ensure internal validity however the survey could also be repeated in other areas of the country and in other countries to compare findings and assess external validity [11]. The results here may also be further analysed in relation to participants from practices of high, medium and low research activity and whether barriers and facilitators differ depending on this activity. Further analysis could also be carried out to differentiate research activity in relation to experience and relate this back to previous literature findings.

One of the recommendations of this study is piloting the time taken to take part in research. A long-term study could look at whether this activity had any influence over staff’s decisions to take part in research. This is also true of improved communications between research teams and primary care. Research into time taken to complete research activity in primary care in relation to money paid for time could provide potential evidence of underfunding. This could then be used to make a case for increased standardised costs providing fair renumeration for time taken to complete research.

## Conclusion

Multiple barriers and facilitators exist to primary care staff taking part in research. Clear, consistent communication at all stages plays a key role. This should begin at the design phase, with input from primary care staff and processes designed to be as straightforward and aligned with existing practice as possible. Opportunities to take part should be communicated clearly and regularly so those who are interested can easily engage. Ongoing two-way communication during the study can help address issues early, and feedback after the study helps staff understand the impact of their contribution and feel their efforts were worthwhile. Basic research training for all staff would improve understanding of processes, ethics and improve confidence. Supporting research in primary care is essential for progress and improved patient care.

This survey was the first to be completed after the COVID-19 pandemic and found staff were more likely to take part in research where there are clear benefits to patients. However, high workloads and inaccurate estimates of time commitments were significant barriers. Staff reported limited confidence or knowledge around research and some felt that topics were not always relevant to their roles. Administrative support helped reduce the burden of research while concerns about ethics and data security showed a lack of familiarity with research protocols from staff. Research was often seen as separate from core clinical work. Research literacy was found to be lower than previous studies although higher for those who were enrolled in further or higher education.

A clear set of recommendations have been produced as a result of this survey. Communication should be improved at all stages with greater collaboration between research teams and primary care during protocol development to ensure feasibility. Study materials should clearly outline patient benefits, time commitments and confirm ethical approval. Routine feedback should be offered to participating staff. It may also be valuable to include research literacy in appraisal processes and develop funded research roles with protected time for research. Further analysis and qualitative research will build a more comprehensive picture but these recommendations offer a solid foundation for reducing barriers and supporting research engagement in primary care.

## Supporting information

S1 Appendix Appendix 1 Participant information sheet and survey. (DOCX)
