# Supplementary material for: A survey of barriers and facilitators to primary care staff conducting research
Source: PLoS One. 2025 Nov 25;20(11):e0334892. doi: 10.1371/journal.pone.0334892 (PMC12646422; doi:10.1371/journal.pone.0334892)
Supplement: S1 Appendix — (DOCX) [file pone.0334892.s001.docx]

**Appendix 1 Participant information sheet and survey**

You are being invited to participate in a research study about barriers and facilitators to primary care staff taking part in research. This study is being carried out by Dr Zoe Edwards, a trainee Advanced Clinical Practitioner from Affinity Care PCN, as part of her MSc in advanced clinical practice.

The purpose of this research study is to carry out an area-wide survey of staff working in primary care looking at your previous and current involvement in research and what helps and hinders this. The survey will take you approximately 5 minutes to complete and it doesn’t matter if you have never taken part in research or have been involved many times. We are looking at views of receptionists, managers and clinicians.

We will use this data to identify some of the barriers and use the things staff have found useful to improve participation in research. We will use Excel and Word to collate data which will be anonymised before subsequent publications to ensure the confidentiality of participants and organisations are protected. Your participation in this study is entirely voluntary and by submitting this questionnaire you are consenting to participate. You do not have to answer any questions you do not want to.

Ethical approval for the study has been granted by the Chair of the Humanities, Social and Health Sciences Research Ethics Panel at the University of Bradford on 8th August 2023.

We believe there are no known risks associated with this research study; however, as with any online related activity the risk of a breach is always possible. We ask for postcodes of workplaces to ascertain where practices are in the Bradford area and to map against research activity in that area. We will not share any information supplied with your workplace or anyone else. Only anonymised data will be published. We will minimise any risks by using password protected computers and personal data will be destroyed according to University of Bradford policy.

If you have any questions about the study, please contact Zoe Edwards on [Z.Edwards@Bradford.ac.uk](mailto:Z.Edwards@Bradford.ac.uk).

You can also contact the research supervisor, Michael Tatterton, via email: [m.tatterton@bradford.ac.uk](mailto:m.tatterton@bradford.ac.uk)

Thank you for taking time to complete the survey.

Version 3   9.8.23

1. **Do you give consent to take part in this survey?**

Yes

No – unfortunately you are not able to take part in this survey. Thank you for your time

1. **Please give the postcode where you work e.g. BD18.**

This will only be used to link your answers to the research activity in your area. We will not pass this information back to the practice in which you work, and it will remain anonymous.

1. **Do you work in an administrative, managerial or clinical role in a GP practice in the Bradford area?**

Yes

No – unfortunately you are not eligible to take part in this study. Thank you for your time.

1. **What is your sex?**

M/F/Prefer not to say

1. **What is your role?**

Administration

Advanced Clinical Practitioner

GP partner

GP locum

GP salaried

Healthcare Assistant

Manager

Nurse

Pharmacist

Physician Associate

Recpetion/care navigator

Other (please specify)

1. **How long have you been in your role?**

0-12 months

1-2 years

3-5 years

5-10 years

10 years plus

1. **What is your highest qualification?**

No qualifications

GCSE/O levels

A Levels

College qualifications – please specify………………….

Undergraduate degree

Postgraduate degree

Other – please specify………………………………………….

1. **Are you currently enrolled in any further/higher education?**

Yes - If so, please state qualification……………………………

No

1. **Before completing this survey have you ever been asked to take part in research or assist with research in your current role?**

Yes – I have been asked in an administrative role

Yes – I have been asked to be interviewed

Yes – I have been asked to be involved in a study

Yes – I have been asked to complete a survey

No

Unsure

Other (please specify)

1. **If you have been asked, how many times have you been asked?**

1-2 times

3-5 times

6 plus times

I have never been asked

1. **How many times have you taken part?**

1-2 times

3-4 times

5 or more times

1. **Thinking about you taking part in research, please rank the following in how much they have or would help you take part (1 would be most helpful, 15 least helpful).**

If I am asked to take part in fewer studies

If I have adequate training for the study

If I have clear directions on what is involved

If I have general training in research

If I have more administrative help

If I have properly allocated time

If involvement was brief

If I know exactly how much time involvement will take

If I know it would make a difference to my colleagues

If I know it would make a difference to my patients

If it was simple to take part

If I were to get personal reward

If I were to be contacted about the outcomes of the study

If the topic interests me

If we were to get team reward

Are there any other things which may help you take part

1. **Thinking about you taking part in research, please rank the following in how much they would or do prevent you from taking part in research (1 is the biggest barrier or problem and 13 is the smallest barrier or problem).**

I cannot fit anything extra in

I don’t feel it is an important topic

I don’t feel my involvement will make any difference

I don’t feel the research is suitable/relevant for our patients

I don’t see what impact it will have

I don’t trust the research team

I feel I would be doing it half-heartedly

I feel other people will be more useful to the research

It is not something I HAVE to doI think the patients would not like to take part

I worry about how much work it will be

I worry about security of patient data

I worry I don’t have the right knowledge to take part

Are there any other barriers you feel?

1. **This section is for clinicians (eg nurses, doctors, pharmacists, ACPs etc). Are you a clinician?**

Yes

No. Thank you for taking part. You have now finished the survey. Please press finish below.

1. **How long is it since you qualified as a healthcare professional?**

0-12 months

1-2 years

3-5 years

6-10 years

11-20 years

11-20 years

21 years or more.

Not applicable as not a clinician

1. **Is research discussed as part of your annual appraisal?**

Yes, routinely

Sometimes

Only if I bring it up

No

I haven’t had an annual appraisal

Not applicable as not a clinician

1. **Thinking about your current involvement in research, please state which best suits you from the following (we know that you may not be involved in any and this is not a problems).**

I would need help to get knowledge from evidence-based research and apply it to practice

I gain new knowledge from evidence or research and apply it to practice

I share awareness of new evidence/research with colleagues, patients and the public and challenge practice to improve care

I use research findings to support change and service development to address clinical challenges (eg contributes to established clinical networks, journal clubs, literature reviews, development of local/national policies)

I actively use own research with the support of others or supports the delivery of research and disseminates research outcomes locally??

I lead the generation of new knowledge through research (eg actively develops and leads) clinical research, engages the research collaborators, obtain research funding, disseminates research nationally/internationally

Not applicable as not a clinician

Thank you for taking part in this survey
